# 

**Published:** 1882-01

**Authors:** 


					﻿NEW BOOKS.
The following works are promised by Duncan Brothers, Chicago:
Text Book of Materia Medica. By A. C. Cowperthwaite, Ph. D., M. D.,
Professor of Materia Medica, etc., Iowa University, Homoeopathic Depart-
ment. A second edition of this popular work is being revised and enlarged,
and will soon be ready.
The Heart and Its Diseases, and their Homoeopathic Treatment.
By W. P. Armstrong, M. D., formerly Lecturer on Diseases of the Heart,
etc., etc.
Diseases of the Urinary Organs. By Chas. Adams, A. M., M. D., Pro-
fessor of Surgery, Chicago Homoeopathic College.
The United States Homoeopathic Pharmacopcea. A second edition
of this work is fast approaching completion.
Angeld on Diseases of the Eye. Sixth edition is promised by Boericke
& Tafel.
THE AMERICAN INSTITUTE.
The next session (June 13th, 1882) of the American Institute, will be held
at Indianapolis, Ind. O. S. Runnels, M. D., is Chairman of the Local Com-
mittee of Arrangements. Change of place is made by order of the Executive
Committee, as it was found to be inexpedient to meet in Richmond, Va.
CONTRIBUTORS:
Drs. H. C. Allen, T. F. Allen, Edward Bayard, J. B. Bell, E. W. Berridge,
Geo. H. Clark, A. C. Co wperth waite, Ad. Fellger, Win. J. Guernsey,
W. A. Hawley, John Hall, W. M. James, W. II. Jenny, Ad.. Lippe,
> V P- Lippe, Malcolm MacFarlan, Thos. Moore, C. F. Nichols,
C. Pearson, T. F. Pomeroy, Leila A. RenDell, C. Carle-
ton Smith, P. P. Wells, Wm. P. Wesselhoeft,
David Wilson, (London), T. P. Wilson,
and many others^
NOTICE.
All articles for publication, books for review, business communications, etc.,
must be sent to Editor, 3109 Chestnut Street, Philadelphia.
Subscribers who have not paid their Subscrip-
tions will please give the matter their prompt
attention. Subscriptions for Volume II.
must be paid by February 1st.
. Authors of accepted papers are furnished with copies of the
number in which their articles appear; application for such copies
to be made when sending papers. Any irregularity in the receipt
of this Journal should be promptly reported to Editor.
Wanted—Copies of the January (1881) number. Thirty cents
paid at this office, for sound copies.
				

